# The Health-Related Consequences of Gender-Based Violence Against Women in South Africa

**DOI:** 10.3390/ijerph23030298

**Published:** 2026-02-27

**Authors:** Andrew Enaifoghe, Ayobami Precious Adekola

**Affiliations:** 1 Department of Public Administration, Faculty of Commerce, University of Zululand, KwaDlangezwa 3886, South Africa; 2 Department of Gender and Sexuality Studies, College of Human Sciences, University of South Africa, Pretoria 0002, South Africa; adekoap@unisa.ac.za

**Keywords:** domestic violence, health, gender-based violence, STIs, cultural beliefs

## Abstract

**Highlights:**

**Public health relevance—How does this work relate to a public health issue?**

- Gender-based violence (GBV) against women in South Africa is a major public health problem due to its widespread occurrence and serious health implications.
- GBV relates to a higher incidence of physical injuries, mental health disorders, and sexually transmitted infections, such as HIV, all of which are a burden on the healthcare system.

**Public health significance—Why is this work of significance to public health?**

- Understanding the health effects of GBV is key to creating effective strategies to reduce morbidity and mortality in affected women.
- The study focuses on the nexus of violence and health, emphasizing the importance of integrated healthcare and social support systems.

**Public health implications—What are the key implications or messages for practitioners, policy makers, and/or researchers in public health?**

- Practitioners should use trauma-informed care and screening processes to identify and support GBV survivors in clinical settings.
- Policymakers and academics must prioritize GBV prevention initiatives and provide resources for comprehensive health services that address both the physical and psychological consequences.

**Abstract:**

Purpose: The study explores the health-related consequences of gender-based violence against women in South Africa. Accordingly, gender-based violence (GBV) has become a serious and pervasive issue in South Africa, affecting practically every aspect of life. Gender-based violence (GBV) persists as a widespread public health emergency in South Africa, disproportionately impacting women across various socio-economic and cultural contexts. This study examines the many health-related effects of gender-based violence, utilising both quantitative data from healthcare facilities and qualitative insights from survivor accounts. The results indicate a significant association between gender-based violence and a heightened prevalence of chronic medical ailments, including hypertension, reproductive health issues, and gastrointestinal diseases. The study also emphasizes a notable increase in mental health illnesses, such as sadness, anxiety, and post-traumatic stress disorder (PTSD), among survivors. The research reveals increasing patterns of intergenerational health effects, indicating that offspring of GBV survivors have increased risks of emotional and behavioural issues. These observations highlight the pressing necessity for cohesive health and social support systems, legislative change, and community-based interventions to mitigate the enduring health impact of gender-based violence on women in South Africa.

## 1. Introduction

Gender-based violence (GBV) against women is a widespread problem in South Africa, sometimes labelled the “rape capital of the world” because of its very high incidence of sexual assault, female murder, and domestic abuse [1]. This violence has significant health implications, including women’s physical, emotional, and reproductive well-being. The repercussions transcend acute physical injuries, leading to enduring mental health disorders like despair, anxiety, and post-traumatic stress disorder (PTSD). Moreover, gender-based violence (GBV) substantially exacerbates the HIV/AIDS epidemic, as women subjected to sexual assault have an elevated risk of HIV infection [2].

The policy ramifications of gender-based violence in South Africa are similarly substantial. Notwithstanding extensive legislative frameworks designed to prevent violence and assist survivors, the execution and efficacy of these laws remain constrained. Progress is hampered by pervasive patriarchal attitudes, pervasive poverty, and inadequate support services. To address these underlying issues, effective policy solutions must include strategies like teaching boys and men about gender equality, empowering women through support and self-defence initiatives, and making sure that government activities are closely monitored and held accountable [3,4].

Developing effective strategies to reduce gender-based violence (GBV) and support affected women requires an understanding of the health-related effects of GBV and the corresponding policy implications in South Africa. The study investigates how gender-based violence against women in South Africa affects their health. As a result, gender-based violence (GBV) has grown to be a significant and widespread problem in South Africa, impacting almost every facet of daily life. to compile information on the health effects and policy implications of gender-based violence against women in South Africa.

### 1.1. Rationale for the Research

With prevalence rates among the highest in the world, gender-based violence (GBV) continues to be one of the most urgent public health and human rights issues in South Africa. Despite decades of policy creation and lobbying, including the National Strategic Plan on Gender-Based Violence and Femicide (2020–2030), health systems still have challenges integrating GBV response into routine care, and implementation gaps still exist. Though these findings are dispersed across disciplines and time periods, recent data connects GBV to important health outcomes like HIV infection, poor maternal health, and mental health issues. For several reasons, this review is now required:

Growing GBV Epidemic: GBV incidence in South Africa increased both during and after the COVID-19 epidemic, underscoring systemic weaknesses and the pressing need for health-sector accountability [5]. Global Alignment and Policy Momentum: In accordance with WHO’s Global Plan of Action to Strengthen Health Systems to Address Violence Against Women, the nation is now implementing the NSP GBVF. A thorough synthesis of the effects on health will direct resource allocation and inform evidence-based policy.

The Need for Integration: GBV interventions are not systematically integrated into current HIV, maternal health, and mental health initiatives. Practical advice for integrating GBV response into clinical practice is given by this review.

Innovation and Research Gaps: The efficacy of GBV interventions in health systems, integration models, and cost-effectiveness assessments is not well documented. In order to determine future research and funding priorities, it is imperative that these gaps be identified immediately.

### 1.2. Search Period

A comprehensive literature search was conducted covering 15 years from 1 January 2009 to 31 December 2024. This date range was selected to capture both historical and contemporary evidence on the health-related consequences of gender-based violence (GBV) against women in South Africa, including the most recent peer-reviewed findings available at the time of the search.

## 2. Research Methodology

This study is qualitative research that primarily collected its data through secondary sources. The study analyzed the data collected through thematic content analysis. The research reviews a substantial amount of literature in the existing body of knowledge, allowing researchers to study, consult, and make sense of written materials or documents available in the public or private domain [6]. The review ensures that the findings are grounded in credible and relevant evidence through the application of systematic search methodologies, inclusion and exclusion criteria, and quality evaluation instruments. The SLR also makes it easier to combine different points of view, like academic theories, policy papers, and reports from the community [7,8]. This enhances the analysis and bolsters conclusions derived from evidence. This study aimed to comprehensively analyze the existing literature regarding the dynamics of power distribution through community engagement and its effects on governance in South Africa.

To accomplish this, the research utilized the Systematic Quantitative Assessment Technique (SQAT), a methodological framework established by scholars [9] The SQAT method has five main steps: (1) clarifying the research topic, (2) developing research questions, (3) selecting appropriate keywords, (4) locating and searching databases, and (5) reviewing and evaluating publications [9], as outlined in Table 1.

Table 1 presents the method applied to collect the secondary data used for the study. Table 2 presents the inclusion and exclusion criteria for the data used for the study.

Table 3 shows the methods of eligibility criteria and study selection.

Table 4 shows the search strategy adopted for the systematic review process. Due to the study’s nature, secondary data was the main source of information. These documents were chosen for this study because they were relevant, trustworthy, and helped us understand how power is shared in participatory governance. Using secondary sources is both effective and morally acceptable, particularly when the collection of primary data is limited by time, access, or resources. It also makes it possible to cover a wider range of times and places, which is important for understanding problems with systemic governance [10].

Relevant literature pertaining to the subject of research was identified utilizing the Scopus, Web of Science, and Google Scholar search engines. The extensive information they supplied rendered these search engines superior to their competitors. The study endeavour included sophisticated keyword searches across many databases for data collecting in search engines from 2014 to 2024 to gather relevant information for the study. Acquiring the latest data on the matter is projected to take ten (10) years. Nevertheless, other studies beyond the specified year ranges were deemed relevant.

### Exclusion and Inclusion Criteria

Exclusion and inclusion criteria were established in accordance with the study’s objectives and subject matter to choose relevant data for the inquiry. Data from magazines, newspapers, and the internet was integrated with excerpts from pertinent peer-reviewed publications related to the subject. The research questions and issues pertaining to the topics were established after a comprehensive study of pertinent publications. Data was examined through developing themes that guided the investigation.

## 3. Conceptualisation of Gender-Based Violence

### 3.1. Gender-Based Violence on the Victim’s Health

Literture suggestes that the survivors of GBV are left to deal with various health and psychosocial issues, which may have long-term negative impacts on their quality of life and livelihood [10,11,12,13]. In South Africa, women have been disproportionately affected by the rising incidence of GBV [14,15,16,17]. Moreover, GBV can only be addressed through a synergized, multisectoral response. In recent years, the international community has taken some timid but significant efforts in bringing more attention to the issue of gender-based abuse [18].

Several United Nations committees, including the Commission on the Status of Women, the Economic and Social Council, and the Committee on Crime Prevention and Control, have passed resolutions condemning violence against women [19]. The Organization of American States is currently negotiating a Pan-American pact against violence against women [20]. This international emphasis follows more than two decades of global activism by women’s groups to resist gender-based assault.

We argue that by understanding the impact of GBV on the health and wellness of women, relevant stakeholders will provoke an enhanced and effective response from the health sector toward addressing the health-related consequences of the violence being perpetrated against women [19,20,21]. Likewise, Rivara et al. indicated that health-related consequences are not only limited to the people at the receiving end of GBV but also extend to perpetrators and the communities where it happens [22,23,24,25].

The authors maintain that various forms of GBV, such as intimate partner violence (IPV) and sexual violence, should be treated as public health issues since they adversely affect the health and wellbeing of the victims. Scholars assert that all forms of violence have health-related consequences [26]. Health-related impacts of GBV could affect the victims mentally, leading to depression, sleep disorder, anxiety, post-traumatic stress disorder (PTSD), and substance abuse [27,28]. This was supported by the findings of other scholars who revealed that women who suffered from GBV are more likely to experience PTSD, depression, and anxiety [29]. The literature analysis revealed that apart from instant death, GBV effects could both be short-term and long-term.

A report by United States Office on Women’s Health in 2022 indicated that short-term health-related consequences of GBV include grievous bodily harm and physical injury, sexually transmitted infections (STIs), including HIV, unplanned pregnancy, vaginal bleeding or pelvic pain, and insomnia or nightmares [30]. The same report highlighted arthritis, asthma, chronic pain, digestive problems, cardiac problems, irritable bowel syndrome, migraine, sexual intercourse trauma, stress, and problems with the immune system as long-term health-related consequences of GBV.

Mosavel et al. concur that GBV has a variety of long-term consequences for adolescent girls and women. Long-term consequences include suicidal ideation, an increased risk of obesity, and risky sexual and health behaviours [31]. As a result, the authors proposed that GBV be addressed in the context of health. Likewise, a 2021 WHO report agreed that providing necessary medical and healthcare interventions to GBV victims is critical to addressing the short- and long-term effects of GBV on health, such as physical and mental health, as well as providing victims with psychosocial support [32].

Besides negative consequences for the victims, GBV puts pressure on the public health sector because it must meet the growing demand for health services from victims of physical, mental, and emotional abuse. While several studies have alluded to the health-related impacts of GBV, there is a knowledge gap as to the extent to which these study findings have been translated to health policies and practices in South Africa. Hence, the researchers were prompted to conduct this study.

The United Nations define violence against women as “any act of gender-based violence that causes or is likely to cause physical, sexual, or mental harm or suffering to women, including threats of such acts, coercion, or arbitrary deprivation of liberty, whether occurring in public or private life” [33]. Physical aggressiveness, sexual coercion, psychological abuse, and controlling behaviours are examples of intimate relationship violence.

This includes rape which is described as “the physically forced or otherwise coerced penetration of the vulva or anus with a penis, other bodily part, or object,” as well as “attempted rape, unwelcome sexual touching, and other non-contact forms [34].” Gender-based violence (GBV) is both a cause and a result of gender disparities [34]. It refers to a variety of violent crimes performed mostly by males against females in the context of women and girls’ lower place in society, and it frequently serves to maintain this uneven balance [35].

Despite a large amount of research on gender-based violence (GBV) in South Africa, there is still no thorough, integrative review that focusses on the entire range of health-related effects of GBV among South African women during the past 15 years. A fragmented knowledge of the multifaceted health burden results from existing reviews’ tendency to focus on individual aspects of violence, particular outcomes, or more comprehensive regional analyses [36]. This fragmentation is demonstrated by several recent reviews:

A 2024 scoping analysis looked at trauma and injuries caused by sexual and gender-based violence in sub-Saharan Africa, but it did not offer a comprehensive evaluation of the effects on mental, reproductive, and chronic health that are unique to South Africa. Women’s experiences seeking care services following sexual violence were examined in a 2023 systematic review, although it only looked at service-seeking behaviour and not the entire health outcome profile.

Although they provide useful information, broader regional evaluations and meta-analyses—such as those looking at the incidence of GBV in 25 SSA countries or the pooled prevalence of intimate partner violence—do not isolate South African health implications or assess how national health outcomes have changed over time. Although they offer rich epidemiological data, national evaluations like the 2022 South African GBV baseline survey do not summarize the health effects documented in clinical, psychological, and public health studies.

### 3.2. Knowledge Gap

- Together, these studies verify that previous evaluations do either one of these two things:
- Concentrate on just one type of GBV, such as sexual violence, only look at a small portion of health outcomes (such as accidents or experiences requesting assistance);
- Combine several African nations without a synthesis unique to South Africa, or focus on causes and prevalence rather than health effects.

Nevertheless, despite South Africa’s internationally reported high incidence of GBV, no review has yet fully compiled and critically analyzed the various physical, mental, reproductive, chronic, and social-health effects of GBV specifically for women in the nation.

### 3.3. Thematic Review of the Literature

1. Consequences of Physical Health

Gender-based violence (GBV) perpetrated against women in South Africa results in significant physical health complications. Research indicates that victims frequently endure injuries that vary from contusions and fractures to more grave conditions such as internal hemorrhaging and persistent pain [35]. Moreover, gender-based violence heightens the risk of sexually transmitted infections (STIs). The physical trauma endured by survivors can result in enduring consequences, affecting their overall health and quality of life.

2. Psychological Health Implications

The psychological effects of gender-based violence are significant. Women subjected to gender-based violence are at an elevated risk of developing mental health disorders, including depression, anxiety, and post-traumatic stress disorder (PTSD). Psychological trauma may result in suicidal ideation and substance abuse as maladaptive coping strategies. Research indicates that the mental health ramifications of gender-based violence are frequently underreported and insufficiently addressed in healthcare environments [9].

3. Consequences of Reproductive Health

Gender-based violence has profound consequences for women’s reproductive health. Survivors of sexual violence may encounter unintended pregnancies, childbirth complications, and reproductive tract infections [37]. The apprehension and stigma linked to gender-based violence can deter women from pursuing essential reproductive health services, thereby worsening their health conditions. Findings shows a correlation between gender-based violence and elevated maternal mortality rates.

4. Societal and Economic Implications

The social and economic ramifications of gender-based violence are significant. Women subjected to gender-based violence frequently encounter social isolation, stigma, and discrimination. This can lead to loss of employment and financial instability, further entrenching them in cycles of poverty [38]. The economic burden of GBV on society includes healthcare costs, legal expenses, and lost productivity. Addressing these consequences requires comprehensive social support systems and economic empowerment initiatives.

5. Policy Implications

Despite the existence of policies aimed at combating GBV, their implementation in South Africa remains inadequate. Factors such as patriarchal norms, lack of resources, and insufficient training for law enforcement hinder effective policy enforcement. Research suggests that policies should focus on prevention, protection, and support for survivors [39,40]. This includes educational initiatives to challenge gender stereotypes, greater access to healthcare and legal services, and strong monitoring and evaluation procedures.

6. Intersectionality and GBV

An intersectional approach is vital in comprehending GBV. Women from vulnerable populations, endure exacerbated impacts of GBV. Intersectionality underlines the necessity for specialized treatments that address the issues encountered by diverse groups of women [40]. Studies highlight the importance of inclusive policies that cater to the diverse experiences of all women.

## 4. Results and Discussion

### 4.1. Data Presentation and Analysis of the Extracted Data

The following section thematically presents the data analysis and the discussion of findings on the available data collected. According to research, intimate relationship violence and sexual coercion are the most frequent types of GBV worldwide, and these are the types of violence examined in this work [2]. GBV has major health repercussions for women, including murders, suicides, and AIDS-related fatalities, as well as physical injuries, chronic pain syndrome, gastrointestinal illnesses, pregnancy issues, miscarriage, and low birth weight of children [2]. GBV also has major consequences for both established and developing economies, including low productivity and profits, as well as low human and social capital accumulation. Research indicates that violence can harm women’s physical, emotional, sexual, and reproductive health and may increase their chance of contracting HIV in specific situations.

A recent directory released by Santiago-based ISIS International cites 379 unique organizations working in Latin America alone to combat female violence [2,40,41,42]. These 135 local efforts must be backed and strengthened by strong government pledges to prevent violence and assist abuse survivors. Some scholars believe that few governments have taken violence against women seriously up to this point, failing to realize the scope of the problem or its implications for health and development [40,43,44,45]. This reflects intentional denial in part, but it is aided by a dearth of good statistics documenting the prevalence and health repercussions of abuse. Furthermore, ignorance is frequently used as a justification for inaction.

### 4.2. Thematic Analysis

#### 4.2.1. Theme 1: The Social and Health Aspects of Gender-Based Violence (GBV)

A widespread problem in human rights and public health, gender-based violence (GBV) disproportionately impacts women and girls. It includes a range of abusive behaviours that frequently overlap in intimate relationships and domestic contexts, including physical, sexual, emotional, and economic abuse. Because GBV is multifaceted, violence affects societal stability, health, and well-being through intricate paths.

#### 4.2.2. Roots in Structure and Gender

Systemic gender inequality, cultural norms, and power disparities that normalize violence against women are the foundations of gender-based violence. Cycles of violence are reinforced by patriarchal structures that maintain control over women’s autonomy. These dynamics are especially noticeable in situations where women’s access to assistance and their capacity to leave abusive relationships are restricted by social standards and economic dependency.

#### 4.2.3. Health Repercussions

The effects of GBV on health are extensive and complex:

- Physical Health: Physical attacks frequently lead to injuries, long-term discomfort, and incapacity. Sustained stress from abuse has been associated with long-term physical issues such gastrointestinal diseases and hypertension.
- Sexual and Reproductive Health: Through forced intercourse and diminished bargaining power for condom use, GBV increases susceptibility to HIV and other STDs. Additionally, it leads to unsafe abortions, unwanted pregnancies [46], and poor outcomes for mothers.
- Mental Health: Suicidal thoughts, sadness, anxiety, and post-traumatic stress disorder (PTSD) are all caused by psychological abuse and trauma. These long-lasting obstacles to recovery are frequently caused by these mental health aftereffects that last long after physical violence stops.
- Behavioural and Social Outcomes: Adopting unhealthy coping mechanisms, such as substance abuse, might worsen health risks for survivors.

#### 4.2.4. Connections to Socioeconomic Elements

Economic abuse, such as denying women access to jobs or resources, increases their reliance and reduces their ability to flee violence. Initiatives for empowerment and education have demonstrated protective effects against GBV, but poverty and food hardship increase vulnerability.

Table 5 classified the various common types of gender-based violence against women and girls, which include but are not limited to domestic violence (DV), intimate partner violence (IPV), sexual violence (SV), traditional harmful practices, and human trafficking [37,45,47]. The present incidence of gender-based violence throughout the world is critical “because of systematic gender inequity in modern society that disempowers women, girls, and other minority groups in society, and thus stifles their voices so that their experiences are not heard” ([48]; p. 3). As a result, women’s basic human rights can be more readily violated.

Violence against women and girls is a multifaceted problem that is situated within a larger socioeconomic, political, and cultural environment, with traditional norms impacting the possibility of GBV. Usama and Ajay noted that an ecological model, which recognizes the various causes of violence and the interplay of risk factors functioning at the individual, interpersonal, community, and society levels, best describes the primary risk factors for GBV [48]. GBV is more common in situations of political, social, and economic inequity and conflict [50]; it is also found in patriarchal societies with rigid notions of manhood, weak institutions, limited access to information, and poor reinforcement of human rights, as well as societies where violence is socially accepted as a means of resolving inter-personal disputes.

When established gender norms are challenged, female empowerment may temporarily raise GBV. However, living in a society where women are valued and have a higher socioeconomic standing protects against GBV [50]. Poverty and a lack of economic possibilities make males more prone to participate in violence and substance misuse, which raises the risk of GBV [48]. Domestic violence is almost six times more likely when the spouse drinks often as opposed to none at all [50]. The danger of GBV is especially high among prostitutes, where the perpetrators are frequently law enforcement agents; for example, 49 percent of female sex workers in Bangladesh had been raped and 59 percent beaten by the police in the preceding year. Females with impairments are a less clear high-risk category [51].

GBV is likely the most common of all human rights abuses, a persistent and systematic public health concern that affects all socioeconomic and ethnic groups worldwide at a significant cost to the person and society. One in every three women has been physically or sexually mistreated, and one in every five has been raped or the victim of attempted raped throughout their lives [52]. The vast majority of GBV occurs at home, where the victim frequently faces repeated assaults [53]. About 68% of sexual attackers are men known to the victim [49]. While men may be exposed to GBV, the health consequences for women are frequently more severe. From 1993 to 2001, 33% of all female homicide victims in the United States were killed by an intimate partner, versus just 4% of male homicide victims [54]. Studies from poor nations indicate relatively comparable conclusions [55].

#### 4.2.5. Health Systems Repercussions

Health systems are heavily burdened by GBV, necessitating coordinated interventions from mental health, maternity health, and HIV care providers. Both systemic determinants and urgent health needs must be addressed by effective treatments. Dismantling norms that support violence requires community mobilization, gender-transformative programs, and legislative changes.

#### 4.2.6. Theme 2: Prevalence of Gender-Based Violence

The majority of research concentrates on DV, IPV, or SV. GBV is frequently underreported due to measuring issues, the issue’s sensitivity, stigma, and ethical considerations. Most research employs non-standardized methodology, making comparison difficult. Demographic Household Surveys (DHSs) employ sentinel questions and modules that are standardized. The prevalence is significantly greater and closer to the genuine rate. This study indicated that with the usage of components of GBV, the prevalence is significantly larger and closer to the genuine figure. The WHO Multi-country Study on Women’s Health and Domestic Violence Against Women [55] employed a standardized technique to gather IPV data from over 24,000 women from 15 locations in ten countries.

According to the study conducted by scholars in 2020 [56], globally, about one in three women encounter gender-based violence during their lifetime, predominantly in the form of intimate relationship violence or non-partner sexual violence. In South Africa, a substantial percentage of women have encountered physical and/or sexual assault, with one survey indicating that 33.1% of women aged 18 and older have endured physical abuse at some point in their lives [57]. According to the findings of this poll, Zambian women have their first sexual contact approximately a year before they become adults (i.e., at the age of 17) or marry [58].

According to the report, 13% of women aged 25 to 49 years had their first sexual contact by the age of 15, while 58% participate in all sorts of sexual activities by the age of 18 [59]. Furthermore, according to this poll, about 15% of women, 11% of men, and 4% of 15- to 19-year-olds in Zambia are HIV-positive [60,61]. Many people have their first sexual encounter as a consequence of compulsion or violence. In poor nations, slightly more than one-third of births to women under the age of 15 were unplanned [62].

#### 4.2.7. Theme 3: Problem of Gender-Based Violence

Finds shows a large body of evidence that documents GBV’s often severe and long-term impact on human health, including but not limited to the following outcomes (Table 6):

This study indicates that GBV has terrible implications not just for individuals who suffer from it, but also for those who see it, particularly youngsters. Victims of GBV frequently experience intense shame and are ostracized and condemned by family, friends, and society. This frequently exacerbates the negative repercussions of GBV [62]. GBV impairs victims’ dignity, autonomy, and security, as well as overall social and economic growth, which frequently reinforces the gender inequalities.

### 4.3. The Health-Related Consequences of GBV Against Women in South Africa

To comprehend the consequences of violence against women, it is critical to recognize that violence against women can result in long-term physical and mental health issues. Not only do violence and abuse harm the women involved, but also their children, families, and communities. These consequences include injury to an individual’s health, potentially long-term harm to children, and harm to communities, such as lost jobs and homelessness. Violence has both immediate and long-term bodily consequences.

#### 4.3.1. Theme 4: Physical Effects of Sexual Violence

The short-term bodily repercussions of violence might range from small injuries to catastrophic conditions. They might include bruises, wounds, fractured bones, or injury to organs and other bodily components. Some physical injuries are difficult or impossible to detect without the assistance of a doctor or nurse (Table 7).

A physical injury can harm both the woman and her unborn child if they are pregnant. This is also true in certain sexual assault situations [63,64]. Many youngsters who witness domestic violence become victims of physical abuse [65].

#### 4.3.2. Theme 5: Long-Term Effects of Sexual Violence

Violence against women, whether sexual or physical, is associated with a variety of long-term health issues. According to the research findings from Smith et al., conducted in 2017, sexual or physical violence is associated with a variety of long-term health issues [51] (Table 8):

Several women experience mental health issues as a result of an assault. To cope with the repercussions of the violence, some women begin to abuse alcohol or drugs or participate in dangerous activities such as unprotected sex. Sexual assault can also have an impact on a person’s perspective of their own body, leading to poor eating habits or eating disorders [33].

#### 4.3.3. Theme 6: The Mental Health Effects of Violence Against Women

If you have been the victim of a physical or sexual attack, you may experience a variety of feelings. You may have feelings of guilt or humiliation as a result of the attack. Some people try to hide or minimize abuse by hiding injuries and making explanations for the abuser. It is not your fault if you have been physically or sexually attacked or mistreated. Seeking therapy for assault or abuse as soon as possible will help prevent long-term mental health impacts and other health problems. The following are some of the long-term mental health consequences of violence against women (Table 9):

Long-term mental health consequences of violence against women might include closing out others, not wanting to do activities you formerly loved, being unable to trust others, and having low self-esteem [60]. Many women who have been victims of violence cope by taking drugs, drinking alcohol, smoking, or overeating. According to research, almost 90% of women with drug abuse disorders had suffered physical or sexual assault [61]. Substance abuse may make you feel better in the short term, but it will make you feel worse in the long run. You will not be able to forget or overcome the event if you use drugs, alcohol, smoke, or overeat. Violence against women can result in death. More than half of the women murdered each year are killed by an intimate partner [64].

### 4.4. Incorporating Results into South African Clinical Practice

According to the review, one of the main causes of women’s poor health outcomes, such as increased HIV risk, poor maternal health, and mental health morbidity, is gender-based violence (GBV). Although GBV is recognized as a priority by policy frameworks like the National Strategic Plan on Gender-Based Violence and Femicide (NSP GBVF) and the National Health Policy, there is still inconsistency in how these commitments are translated into clinical practice. The South African health system must implement effective, context-specific solutions that integrate GBV response into standard care in order to close this gap.

7. *Regular Risk Assessment and GBV Screening*

Operationalisation: Include GBV screening questions in routine patient intake forms for HIV services, prenatal clinics, and primary healthcare. Make use of WHO-recommended resources that have been modified for regional languages and cultural settings [65]. Teach community health workers and nurses how to perform screening in a sensitive manner while maintaining confidentiality and privacy. To standardize documentation, incorporate electronic prompts into the District Health Information System (DHIS).

8. *Clinical Protocols Informed by Trauma*

Operationalisation: Create and distribute clinical guidelines that address physical injuries, mental health referrals, and post-rape treatment for GBV survivors. Provide emergency contraception and rape kits to facilities, and make sure staff members are educated in compassionate, nonjudgmental treatment.

9. *Including HIV and Maternal Health Services*

Operationalisation: Integrate safety planning and GBV counselling into PMTCT and HIV preventive initiatives. Connect ART adherence support with GBV screening. Employ task-shifting methods to lessen the stress on providers by having lay counsellors offer HIV counselling in addition to GBV support.

10. *Improving Assistance for Mental Health*

Operationalisation: Provide mental health treatments with specific referral channels for GBV survivors in primary care clinics. Develop collaborations with NGOs for trauma counselling and provide community health professionals with basic psychosocial support training.

11. *Referral Networks Across Sectors*

Operationalisation: Using digital platforms and standardized referral forms, establish official connections between medical facilities, social services, and legal assistance. Test “one-stop centres” that provide survivors with a single location for access to legal, medical, and psychosocial services.

12. *Development of Workers*

Operationalisation: Offer ongoing professional development and include GBV modules into nursing and medical curricula. To scale training across rural and urban facilities, use blended learning (online + in-person).

13. *Observation and Assessment*

Operationalisation: For regular reporting and quality enhancement, incorporate GBV indicators into DHIS. Monitor health outcomes, screening rates, and referral uptake to guide adaptive tactics.

### 4.5. Use a Public Health Perspective

GBV is treated as a systemic factor of population health as well as a clinical problem that affects individuals when seen through a public health perspective [66]. In line with WHO’s Global Plan of Action to Strengthen Health Systems to Address Violence Against Women, this strategy places a strong emphasis on prevention, early identification, and integrated care.

## 5. Interpretation of Findings

Intimate partner (physical, sexual, and psychological) violence and sexual violence cause substantial short- and long-term physical, mental, sexual, and reproductive health issues in women. They also have an impact on the victims’ children’s health and well-being [67]. This violence has significant social and economic consequences for women, their families, and society. According to the assessment, gender-based violence (GBV) against women is a public health emergency in South Africa because it is not only widespread but also closely linked to negative health outcomes. Several significant trends show up:

### 5.1. GBV as a Cause of HIV and Risks to Sexual Health

Evidence repeatedly demonstrates that women are more susceptible to HIV and other sexually transmitted illnesses because of intimate partner violence (IPV) and other types of gender-based violence. Relationship power disparities, forced sex, and the inability to negotiate condom use are other mechanisms [66]. This data highlights the need for combined GBV-HIV interventions and is consistent with the high HIV prevalence among women in South Africa. Based on the findings of this study, we argue that there is a need to proactively treat GBV as a public health emergency, which requires comprehensive, responsive, and integrated health policy to guide healthcare delivery for the survivors of GBV. In line with other studies [68], it is critical to mobilize and educate health workers, GBV survivors, and the public on how to access healthcare services through a functional referral system.

This requires training all categories of health workers, from first responders to clinicians at all levels, to identify and support victims of GBV professionally. In line with a study conducted by Enaifoghe et al. [2], we advocate for more resources to be allocated for the training of healthcare workers and support staff, which should be targeted at preventing and managing GBV in the country. Furthermore, the allocated resources should be used to provide more infrastructure and facilities to boost access to counselling, psychosocial support, and physical and mental healthcare services at the community level nationwide [69]. Both the provincial and national health departments should use their facilities as centres to create awareness about GBV as well as advocacy centres for the prevention of GBV.

### 5.2. The Burden of Mental Health

Post-traumatic stress disorder (PTSD), anxiety, and depression are all closely linked to GBV. Long-term obstacles to health-seeking behaviour and treatment adherence are often caused by these mental health aftereffects, which frequently last beyond the direct experience of violence [70]. A significant gap in the availability of mental health services in primary care settings is brought to light by this review. The multisectoral collaboration between the health sector, the criminal justice system, social developments, community leaders, and non-governmental organizations need to be strengthened to be more responsive to the health needs of the victims of GBV. Such collaboration should aim to build GBV prevention awareness at the community level, equip health and allied workers to detect GBV early and provide medical services to address any reproductive, mental, and physical needs of the victims.

### 5.3. Effects on Maternal and Reproductive Health

Negative consequences include miscarriage, stillbirth, and unwanted pregnancies are associated with violence during pregnancy. These results highlight the significance of including psychosocial support into maternal health programs and conducting IPV screenings during prenatal visits. This study discovered that gender-based violence is one of the most common human rights abuses worldwide. It has little regard for social, economic, or national borders. One in every three women may experience physical or sexual abuse in her lifetime, according to estimates [71]. Gender-based violence jeopardizes victims’ health, dignity, security, and autonomy, yet it is cloaked in a culture of silence. The following policy must be put in place to prevent and address situations of gender-based violence in any form, whether as an act of workplace violence or in any employee’s personal life (the policy). Gender-based violence, which includes domestic abuse, sexual assault, and stalking, endangers the lives and safety of millions of individuals throughout the world.

### 5.4. Gaps in the Health System

Health facilities continue to differ in their GBV screening and response practices despite policy pledges. Inadequate care and under-identification of survivors are caused by weak referral mechanisms, a lack of standardized procedures, and inadequate provider training. A protection order, restraining order, or other court order issued in response to gender-based violence. Protection orders may sometimes be imposed as a condition of probation or parole in criminal proceedings, particularly in circumstances involving domestic violence, sexual assault, dating violence, or stalking [72].

### 5.5. Socioeconomic and Structural Mediators

GBV risk is increased and access to health services is hampered by economic reliance, poverty, and deeply ingrained gender stereotypes. These structural factors emphasise the necessity of multi-sectoral strategies that integrate social protection and empowerment initiatives with health interventions [73]. Safety planning refers to the process through which an organisation representative collaborates with a victim to promote the creation of a safety and support plan (safety plan) meant to decrease the risk of victims facing gender-based violence and safeguard the victim’s coworkers.

Crime prevention and criminal justice interventions are critical components of this strategy [74]. Since 2010, UNODC has assisted countries in ensuring that this is done in a victim-centred manner, in accordance with the updated Model Strategies and Practical Measures on the Elimination of Violence Against Women in the Fields of Crime Prevention and Criminal Justice, as well as other related international standards and norms.

### 5.6. Gaps in Research and Evidence

Few studies examine cost-effectiveness or assess how well GBV interventions work within health systems. Additionally, there is little data on integrated models that connect social, legal, and health services. The capacity to scale evidence-based solutions is limited by these shortcomings. The implication is that all the results suggest that GBV is a systemic health determinant that warrants a public health strategy. It is crucial to incorporate GBV screening, trauma-informed care, and mental health assistance into standard clinical practice. Sustainable impact will also depend on addressing structural factors through community-level interventions and policy.

## 6. Conclusions

Examining the health-related effects of gender-based violence (GBV) against women in South Africa and its implications for health policy and practice was the goal of this review. The data shows that GBV is a serious public health concern as well as a human rights violation, as it raises the chance of HIV infection, has a negative impact on maternal outcomes, lowers adherence to HIV treatment, and negatively affects mental health. These results highlight the necessity of multi-sectoral, coordinated interventions in health systems that target the structural causes of violence as well as urgent clinical needs.

Health policy must integrate GBV response into HIV and reproductive health initiatives, improve mental health services, and institutionalize GBV screening in order to have a significant impact. Evidence-based, long-lasting solutions will be ensured by connecting these tactics to WHO guidelines and national health agendas. In South Africa, addressing GBV from a public health perspective is crucial to enhancing health outcomes, lowering disparities, and promoting gender justice.

However, several research gaps remain. Evidence on the effectiveness of policy interventions—such as routine GBV screening and integrated HIV/GBV services—is limited, with few rigorous evaluations of implementation outcomes. Similarly, the integration of health and social services for survivors requires further study to determine optimal models for coordination and sustainability. Additional gaps include the long-term health trajectories of GBV survivors, the impact of economic empowerment programs on health outcomes, and cost-effectiveness analyses of GBV interventions within resource-constrained settings.

Addressing these gaps is essential for informing evidence-based policy and practice. Future research should prioritize longitudinal designs, implementation science approaches, and evaluations of multi-sectoral strategies. Positioning GBV within a public health framework, aligned with WHO guidelines, will strengthen South Africa’s response, improve health outcomes, and advance gender equity.

Findings from this study show that the consequences of GBV result in a rise in morbidity and mortality rates worldwide because of its physical, mental, emotional, and social inflictions on victims. As a result of lifestyle changes, the quality of life has suffered. The study further highlighted that the biggest health consequences could include bodily consequences such as organ loss following an assault, unwanted pregnancies, sexually transmitted infections, and possible death. The findings of this study showed that GBV has a significant negative impact on the physical and mental health of the victims.

### 6.1. Recommendations for Future Research

Future research should focus on longitudinal studies to determine the long-term health implications of GBV. There is also a need for more detailed data on the effectiveness of existing policies and programs. Collaborative efforts between researchers, politicians, and community organizations can lead to more successful methods to combat GBV and help survivors.

#### 6.1.1. Effectiveness of Policy and Programmatic Interventions

Limited empirical data exist on the impact of national GBV policies and health-sector interventions (e.g., routine screening, integrated HIV/GBV services) on health outcomes. Rigorous evaluations using implementation science frameworks are needed.

#### 6.1.2. Integration of Health and Social Services

Research should explore optimal models for linking health facilities with social support systems, legal aid, and shelters. Studies should assess feasibility, sustainability, and cost-effectiveness of multi-sectoral referral networks.

### 6.2. Limitations of the Study

Despite the significance of this research, several limitations must be acknowledged:

- Underreporting of GBV Cases: Due to stigma, fear of retaliation, and lack of trust in law enforcement or healthcare systems, many women do not report incidents of GBV. This underreporting may result in an underestimation of the true prevalence and health impact of GBV.
- Data Availability and Reliability: The study relies on secondary data from health institutions and surveys, which may not consistently document GBV-related health outcomes. Incomplete or inconsistent records can affect the accuracy of findings.
- Geographical and Demographic Constraints: The research may focus on specific regions or populations, limiting the generalizability of the findings to all women in South Africa, especially those in rural or marginalized communities.
- Causality Challenges: Establishing a direct causal link between GBV and specific health outcomes is complex due to the influence of multiple intersecting factors such as poverty, mental health history, and access to healthcare.
- Ethical and Emotional Sensitivities: Engaging with survivors of GBV presents ethical challenges and emotional risks for both participants and researchers. These sensitivities may limit the depth of qualitative data collection.
- Cultural and Social Barriers: Cultural norms and societal attitudes toward gender roles and violence may influence how openly participants discuss their experiences, potentially affecting the authenticity of responses.
- Resource Limitations: Constraints in funding, time, and access to comprehensive datasets may restrict the scope and depth of the research.

## Figures and Tables

**Table 1:** Description and application of SQAT.

Steps	Application in the Study
Define topic	Health-Related Consequences of Gender-Based Violence Against Women.
Formulate research questions	What are the health consequences of GBV against women in South Africa? What are the implications of these consequences for health policies and practices in South Africa?
Identify keywords	“Gender-based violence; Domestic violence, health; violence against women; STIs; cultural beliefs”,
Identify and search databases.	Google Scholar/Scopus/Google/ PubMed/ Scopus/Web of Science/and African Journals Online (AJOL)
Read and assess publications.	A preliminary content scan assessed the literature found during the search. After that, the papers were examined and reviewed for relevance to the discussion topic of the Health-Related Consequences of Gender-Based Violence Against Women and Policy Implications in South Africa.
Use of secondary sources	Using academic databases (like JSTOR, Scopus, and Google Scholar) and government portals to search the literature by keyword. Reviewing documents and analyzing their content to find relevant themes, results, and policy implications.

Source: Author’s own computation.

**Table 2:** Inclusion and exclusion criteria.

Criteria	Inclusion		Exclusion	
Geographic Focus:	Studies and reports specifically focused on South Africa.		Studies not focused on South Africa or lacking contextual relevance.	
Topic Relevance:	Literature addressing the health-related consequences of gender-based violence against women in South Africa. The inclusion criteria were centred on the following types of research:		Literature that discusses the health-related consequences of gender-based violence against women in South Africa.	
		Discussion about health-related consequences of gender-based violence against women in the context of South Africa. Addresses health-related consequences of gender-based violence against women in South Africa. Offer theoretical or empirical perspectives that are pertinent to practice and policy.		Studies conducted outside of South Africa or that had no bearing on health-related issues of GBV were excluded.
Publication Type:	Peer-reviewed journal articles, government reports, NGO publications, international development agency documents, and credible media sources.		Opinion pieces or blog posts lacking credible sources or institutional backing.	
Time Frame:	Literature published between 2014 and 2025 was chosen to ensure relevance to current socio-economic conditions. Other older studies were added based on their relevance.		Duplicate studies or reports already included in other reviewed sources.	
Language:	Publications in English.		Publications in languages other than English.	
Accessibility:	Full-text availability for review and analysis.			

Source: Author’s own computation.

**Table 3:** Methods: eligibility criteria and study selection.

Criteria	Application
Justification for Time Frame	The 2000–2025 window was chosen to reflect the post-apartheid era, during which significant legislative and policy reforms addressing GBV were implemented. References to studies outside this period were included only for contextual background and were not part of the systematic review dataset.
Search Strategy	A comprehensive search was conducted across the following databases: PubMed, Scopus, Web of Science, and African Journals Online (AJOL). The search combined keywords and Boolean operators related to the following: Population: “women” OR “girls” Exposure: “gender-based violence” OR “intimate partner violence” OR “sexual violence” Outcome: “health consequences” OR “mental health” OR “physical health” Location: “South Africa”
Study Selection Process	All identified records were imported into EndNote for deduplication. Screening occurred in two stages: Title and Abstract Screening: Two independent reviewers assessed relevance based on inclusion/exclusion criteria. Full-Text Review: Eligible articles underwent detailed evaluation. Discrepancies were resolved through discussion or a third reviewer.

**Table 4:** Search strategy for systematic review.

Database	Search String	Date of Search	Filters Applied
PubMed	(“gender-based violence” OR “intimate partner violence” OR “sexual violence”) AND (“health consequences” OR “mental health” OR “physical health”) AND (“South Africa”) AND (“women” OR “girls”)	20 March 2025	English; 2000–2025
Scopus	TITLE-ABS-KEY(“gender-based violence” OR “intimate partner violence” OR “sexual violence”) AND TITLE-ABS-KEY(“health consequences” OR “mental health” OR “physical health”) AND TITLE-ABS-KEY(“South Africa”)	20 March 2025	English; 2000–2025
Web of Science	TS = (“gender-based violence” OR “intimate partner violence” OR “sexual violence”) AND TS = (“health consequences” OR “mental health” OR “physical health”) AND TS = (“South Africa”)	20 March 2025	English; 2000–2025
AJOL	(“gender-based violence” OR “intimate partner violence” OR “sexual violence”) AND (“health consequences” OR “mental health” OR “physical health”) AND (“South Africa”)	20 March 2025	English; 2000–2025
Grey Literature	Government reports, NGO publications, and policy documents were searched using Google and institutional repositories with the keywords “GBV health outcomes South Africa”	20 March 2025	English; 2000–2025

**Table 5:** Gender-based violence ^1^.

	Type of GBV	Description—Meaning
Gender-Based Violence (GBV)	Domestic Violence (DV)	An intra-family member perpetrates this.
	Intimate Partner Violence (IPV)	This includes physical, sexual, or psychological harm by a current or former partner or spouse.
	Sexual Violence (SV)	Includes rape, sexual abuse, forced pregnancies, and prostitution.
	Traditional harmful practices	This includes female genital mutilation (FGM), honour killing, and dowry-related violence.
	Human trafficking	This is referred to as the recruitment, transportation, transfer, harbouring, or receipt of people through force, fraud, coercion, or deception, to exploit them for profit [37].

Source: ^1^ Saferspaces (2019) [49]. Gender-based violence in South Africa; https://www.worldbank.org/en/topic/socialsustainability/brief/violence-against-women-and-girls (accessed on 2 February 2025). Compiled by the authors (Enaifoghe & Adekola).

**Table 6:** The main public health challenge in South Africa.

The Main Public Health Challenge	**Results of GBV**
	Fatal outcomes
	Acute and chronic physical injuries and disabilities
	Serious mental health problems and behavioural deviations that Increase the risk of subsequent victimization
	Gynecological disorders
	Unwanted pregnancies, obstetric complications, and HIV/AIDS

Source: compiled by the authors.

**Table 7:** Physical effects of sexual violence.

Short-term physical effects of sexual violence	Vaginal bleeding or pelvic pain
	Unwanted pregnancy
	Sexually transmitted infections (STIs)
	HIV/AIDS
	Trouble sleeping or nightmares

Source: compiled by the authors.

**Table 8:** The long-term health problems.

Long-term physical effects of sexual violence	Arthritis
	Asthma
	Chronic pain
	Digestive problems such as stomach ulcers
	Heart problems
	Irritable bowel syndrome
	Nightmares and problems sleeping
	Migraine headaches
	Sexual problems such as pain during sex
	Stress
	Problems with the immune system

Source: compiled by the authors.

**Table 9:** Long-term mental health effects.

Post-traumatic stress disorder (PTSD)		This can be the outcome of trauma or a surprising or frightening encounter, such as sexual assault or physical abuse [65]. The victim may be easily startled, tense or irritable, have trouble sleeping, or have furious outbursts. The victim may also experience difficulty remembering things or have unfavourable views about themselves or others.
Depression		Depression is a prevalent mental illness. It is believed that 5% of individuals worldwide suffer from depression. Depression is a primary cause of disability globally and a significant contributor to the global illness burden. Depression affects more women than men. Suicide can be caused by depression. Treatment is available for mild, moderate, and severe depression.
Anxiety		This can range from general uneasiness about everything to a sudden assault of extreme panic. Anxiety might worsen over time and disrupt your daily life.
Other effects		Victim closing others off, refusing to do activities you used to love, being unable to trust others, and having poor self-esteem [64].

Source: compiled by the authors.

## Data Availability

The data supporting the findings of this study are within the paper. No new data were generated.
